# Economic growth and household energy footprint inequality in China

**DOI:** 10.1371/journal.pone.0282300

**Published:** 2023-03-01

**Authors:** Qiaoqiao Zhu, Xiaowen Sang, Zhengbo Li

**Affiliations:** 1 Xinjiang Innovation Management Research Center, Xinjiang University, Urumqi, China; 2 School of Economics and Management, Xinjiang University, Urumqi, China; Institute for Advanced Sustainability Studies, GERMANY

## Abstract

There are significant differences in energy footprints among individual households. This study uses an environmentally extended input-output approach to estimate the per capita household energy footprint (PCHEF) of 10 different income groups in China’s 30 provinces and analyzes the heterogeneity of household consumption categories, and finally measures the energy equality of households in each province by measuring the energy footprint Gini coefficient (EF-Gini). It is found that the energy footprint of the top 10% income households accounted for about 22% of the national energy footprint in 2017, while the energy footprint of the bottom 40% income households accounted for only 24%. With the growth of China’s economy, energy footprint inequality has declined spatially and temporally. Firstly, wealthier coastal regions have experienced greater convergence in their energy footprint than poorer inland regions. Secondly, China’s household EF-Gini has declined from 0.38 in 2012 to 0.36 in 2017. This study shows that China’s economic growth has not only raised household income levels, but also reduced energy footprint inequality.

## Introduction

The elimination of energy inequalities and sustainable development have become critical issues of global concern, especially after the United Nations Conference on Environment and Development in 2002. In addition, the combination of poverty eradication and clean energy is the focus of the UN Sustainable Development Goals (SDGs) [1, 2]. The energy footprint is the energy consumption directly or indirectly caused by an individual, organization, project, or production process [3]. The energy footprint has been increasingly used to measure the impact of human activities on global climate change [4–6]. The close link among the wealth gap, energy footprint inequality and energy poverty has been demonstrated [7–9]. This study aims to provide policymakers with information to help them understand more about the relationship among poverty, inequality and energy footprint in designating policy measures. Therefore, this paper will measure the energy footprint of different income groups in China.

For developing economies, addressing the two issues of poverty and clean energy will contribute to achieving Sustainable Development Goals. Several studies have shown that economic growth can alleviate energy poverty [10–12]. Firstly, economic growth will increase financial support for using clean energy and technological innovation for energy efficiency and emission reduction in the relevant sectors, which will contribute to a sustainable energy transition in industrialized development at the source [13]. Secondly, economic growth will increase the actual income of economic agents, enhance people’s ability to pay for clean facilities, and improve the energy-poor living conditions of low-income groups so that they can follow a green and low-carbon lifestyle [14]. Thus, the link between economic growth and sustainable energy development is quite strong. It is essential to invest in using clean energy in energy consumption to achieve sustainable economic development. In such a context, an in-depth exploration of the poverty-energy nexus is a significant prospect in sustainable development. Households vary considerably in income levels, consumption patterns, and their contribution to the energy footprint. Household consumption is a necessary consideration when formulating energy policies in countries with a high energy footprint and energy intensive [15]. Therefore, studying inequalities in household energy footprint is significant for achieving the win-win goals of economic growth and energy efficiency.

A large number of studies have measured inequalities in energy consumption at the national level or the regional level within countries. However, studies addressing energy inequalities at the household level are still very limited. The global energy footprint increased by 29.4% from 1995 to 2009, with economic activity being the most crucial driver of the increased energy footprint [16]. In recent years, economic growth has become less dependent on energy [17]. In addition, in developed countries such as Denmark, the United Kingdom, France and the United States, two opposite drivers—reduced energy intensity and increased per capita consumption—lead to changes in the energy footprint [18]. However, there are tremendous inequalities in the international energy footprint. Honglin et al. (2020) studied energy inequalities between and within ten countries in Latin America and the Caribbean (LAC). The results show that the energy footprint of the top 10% income group accounts for 26.3% of the region’s total energy footprint, which is five times larger than the energy footprint of the bottom 10% income group [19]. Household energy consumption includes direct and indirect parts. Energy use in household daily life (coal, gasoline, diesel, liquefied petroleum gas, natural gas, heat, electricity, etc.) is considered as direct energy consumption. Energy consumption in the production, transportation and marketing processes of the sectors providing goods or services is considered indirect energy consumption in the household sector [20]. The inequality in the distribution of the energy footprint varies across goods and services. Energy-intensive goods tend to be more elastic, resulting in a larger energy footprint for higher-income groups [21]. Anne & John (2020) calculated the energy consumption of British households with different incomes in the whole supply chain of goods and services by using the energy-expanded multi-regional input-output model [22]. The study suggests that the lifestyle of high-income households consumes nearly five times more energy than low-income households, but the policy costs paid by high-income households are only 1.9 times higher due to higher levies on energy bills only. Marta et al. (2021) examined the household energy footprint of Nepal, Vietnam, and Zambia [23]. The research found that in all three regions, household energy consumption was much lower for high living standards due to the transition from inefficient biomass-based traditional fuels to efficient modern fuels such as natural gas and electricity.

In an overview of current energy footprint studies, macroscale and mesoscale energy footprint studies, represented by countries and provinces, are at a more mature stage. In contrast, very few articles have studied household energy footprint inequality in depth from a micro view. In addition, research on the relationship between economic growth and the equality of household energy footprint based on microscopic perspectives is even rarer. Based on this, this paper will further extend the above study using multi-regional input-output tables (MRIO) in both time and space. The PCHEF of China’s 30 provinces (excluding Tibet and Hong Kong, Macao, and Taiwan due to data limitations) in 2012 and 2017 are measured. The heterogeneity of the PCHEF of 10 urban and rural income groups and eight categories of consumption expenditures (food, clothing, residence, household facilities, transport, education, health care, and others) are analyzed. The Gini coefficient is used to measure the inequality of household energy footprint. Finally, this paper analyzes the correlation between China’s economic development and the equality of household energy footprint, and discusses how to realize the equality of household energy footprint on the basis of economic development, in order to provide decision-making reference for China to achieve the win-win goal of energy conservation, emission reduction and economic growth.

## Research methods and data sources

In this study, the PCHEF of 10 income groups in 30 regions in China is estimated using an environmentally expanded input-output method. The income groups are derived from the provincial statistical yearbooks and include five urban and five rural groups. The population is evenly divided into five groups from low income to high income: low income, middle and lower income, median income, middle and upper income, and high income. And finally, EF-Gini are calculated to measure the energy inequality of households.

### Data sources and construction of MRIO tables

We use the 2012 and 2017 MRIO tables for China’s 26 provinces and 4 cities, except Tibet, Hong Kong, Macao, and Taiwan (in total, 30 regions), which are obtained from the Carbon Emission Accounts & Datasets (CEADs). CEADs gathers a group of experts from the UK, USA and China to work on China and other emerging economies’ emission accounting methods and applications. All datasets published by CEADs are the results of current research projects funded by National Natural Science Foundation of China, Ministry of Science and Technology of China, Research Councils UK. Relevant data is available on the CEADs website (https://www.ceads.net/). To calculate energy consumption embodied in imports, we connected China’s MRIO tables to the global MRIO table, which are based on the Organization for Economic Co-operation and Development Inter-Country Input-Output Tables (OECD-ICIO). The OECD-ICIO describes international trade connections for 45 industries among 67 economies. China is one of the economies in the OECD-ICIO, so we reformed the OECD-ICIO into 33 sectors and disaggregated the China-related industries in the OECD-ICIO into 30 regions and 33 sectors according to China’s MRIO table. The energy intensity data are obtained from the CEADs database. The family consumption and income data are obtained from the provincial statistical yearbooks in China [24, 25].

### Research methodology

#### Multi-regional input-output model

The input-output method is a quantitative model of economics created by the American economist Wassily Leontief that can analyze the interdependence between different sectors [26]. The environment-extended input-output approach introduces an environmental satellite account into the traditional input-output process to measure the environmental impacts of socio-economic activities [27–29]. We applied an environmentally extended multi-regional input-output model to estimate the PCHEF for 10 different income groups of China’s 30 regions. It has grown to the most mature and comprehensive research technique to determine household carbon footprint in data analysis [30–32]. Still, few studies have applied it to the household energy footprint [33]. The MRIO model uses a linear system of equations to describe the economic linkages between different sectors in different regions. The basic linear equation is:

$$X={\left(I-A\right)}^{-1}F$$ (1)

$$X=\left[\begin{array}{c} x^{1}\\ x^{2}\\ \vdots \\ x^{n} \end{array}\right],A=\left[\begin{array}{cccc} a^{11} & a^{12} & \cdots & a^{1n}\\ a^{21} & a^{22} & \cdots & a^{2n}\\ \vdots & \vdots & \ddots & \vdots \\ a^{n1} & a^{n2} & \cdots & a^{nn} \end{array}\right],F=\left[\begin{array}{cccc} f^{11} & f^{12} & \cdots & f^{1n}\\ f^{21} & f^{22} & \cdots & f^{2n}\\ \vdots & \vdots & \ddots & \vdots \\ f^{n1} & f^{n2} & \cdots & f^{nn} \end{array}\right]$$ (2)

where $X=(x_{i}^{s})$ denotes the total output, $x_{i}^{s}$ denotes the total output of sector *i* in region *s*, *I* is the unit matrix, and ${\left(I-A\right)}^{-1}$ is the Leontief inverse matrix. The technology coefficient submatrix $A^{rs}=(a_{ij}^{rs})$ can be obtained by, where $a_{ij}^{rs}=z_{ij}^{rs}/x_{j}^{s},z_{ij}^{rs}$ denotes the cross-sectoral currency flow from sector *i* in region *r* to sector *j* in region *s* and $x_{j}^{s}$ denotes the total output of sector *j* in region *s*. $F=(f_{i}^{rs})$ denotes the final demand matrix, where $f_{i}^{rs}$ denotes the final demand for goods from sector *i* in the region *s* to sector *i* in the region *r*.

The energy footprint is calculated using environmentally expanded input-output analysis. We refer to the analytical model of Mi et al. (2020) [34], and make corresponding modifications according to the research needs of this paper. Based on the energy intensity (i.e., energy consumption per unit of economic output), the total energy footprint is calculated as follows:

$$E=K{\left(I-A\right)}^{-1}F$$ (3)

where *E* is the total energy footprint and *K* represents the energy intensity of each economic sector in all regions that is expressed by energy consumption per unit of output. In the process of calculating the energy consumption of different industries in each province, there are 20 types of energy involved, including raw coal, cleaned coal, other washed coal, briquettes, coke, coke oven gas, other gas, other coking products, crude oil, gasoline, kerosene, diesel oil, fuel oil, LPG, refinery gas, other petroleum products, natural gas, heat, electricity, other energy. Then, the different energy consumption of each province is converted into standard coal according to the energy conversion coefficient provided by the China Energy Statistical Yearbook. The final demand (i.e., *F*) can be divided into rural household consumption, urban household consumption, government consumption, fixed capital formation, and inventory change. Thus, the household energy footprint is calculated as follows:

$$E_{h}=K{\left(I-A\right)}^{-1}H$$ (4)

where *E*_*h*_ is the PCHEF and *H* is the per capita household consumption column vector, including rural and urban household consumption. Since we use MRIO tables at the provincial level, domestic and imported goods can be further divided into self-production, domestic inflows, and international imports categories. Referring to the consumption category classification method of Ma et al. (2019), the household energy footprint computed by the MRIO model can be grouped into eight consumption categories: food, clothing, residence, household facilities, transport, education, health care, and others [35].

**The EF-Gini.** The Gini coefficient was introduced by the Italian economist Corrado Gini to quantify the level of variation in income distribution [36]. The Gini coefficient varies from 0 to 1, indicating a change in income distribution from perfect equality to perfect inequality [37]. The basic income Gini coefficient is calculated as follows:

$$G=\frac{1}{2\mu N^{2}}\sum_{i=1}^{N}\sum_{j=1}^{N}\left|y_{i}-y_{j}\right|$$ (5)

Where *G* denotes the Gini coefficient. *μ* denotes the expected value of income for each subgroup. *y*_*i*_ and *y*_*j*_ are the mean values of income for the corresponding subgroups. *N* denotes the number of subgroups [38, 39]. Similarly, the EF-Gini can be calculated by replacing the income in the equation with an energy footprint [40].

## Results and analysis

### The PCHEF

At the national level, China’s PCHEF grew by 136%, or 651 tons of standard coal, from 2012 to 2017, with 60% of the growth coming from consumption in urban areas. The affluent eastern region’s PCHEF grew more than the central and western regions. But the western region, which has a relatively poorer economic development, grew more rapidly compared to the central region. Specifically, it increased by 144% in the east, 132% in the west and 127% in the central region. Despite the relative poverty in western China, high-quality economic development in recent years has conspicuously boosted consumption and GDP growth rates in the western region. At the provincial level, the PCHEF have increased in almost all provinces. For example, two of China’s western provinces, Xinjiang and Ningxia, have seen the PCHEF increase by 458% and 433%, respectively. Beijing, Shanghai, and Tianjin, three of China’s most economically developed regions, have seen their PCHEF increase by 102%, 101%, and 126% respectively. Guangdong was the only province with a declining PCHEF, which reduced by 14% between 2012 and 2017. The phenomenon is mainly caused by a decrease in energy intensity (i.e., energy consumption per unit of economic output) and the proportion of energy-intensive household consumption. The carbon Emissions Trading System (ETS) opened in Guangdong in 2013. By standardizing low-carbon management systems, carbon emission trading policies encourage enterprises to pursue progress in energy conservation and emission reduction technologies [41]. More critically, after the launch of the 13th Five-Year Plan in 2016, the transfer of labor-intensive enterprises from developed cities to developing cities stimulated the adjustment and upgrading of Guangdong’s industrial structure, and the energy consumption of industrial production was reduced [42]. In addition, from 2012 to 2017, Guangdong’s household consumption of energy-intensive food and transportation decreased by 4.3 and 3.9 percentage points respectively [43, 44].

### Further analysis of the PCHEF

**Fig 1:**
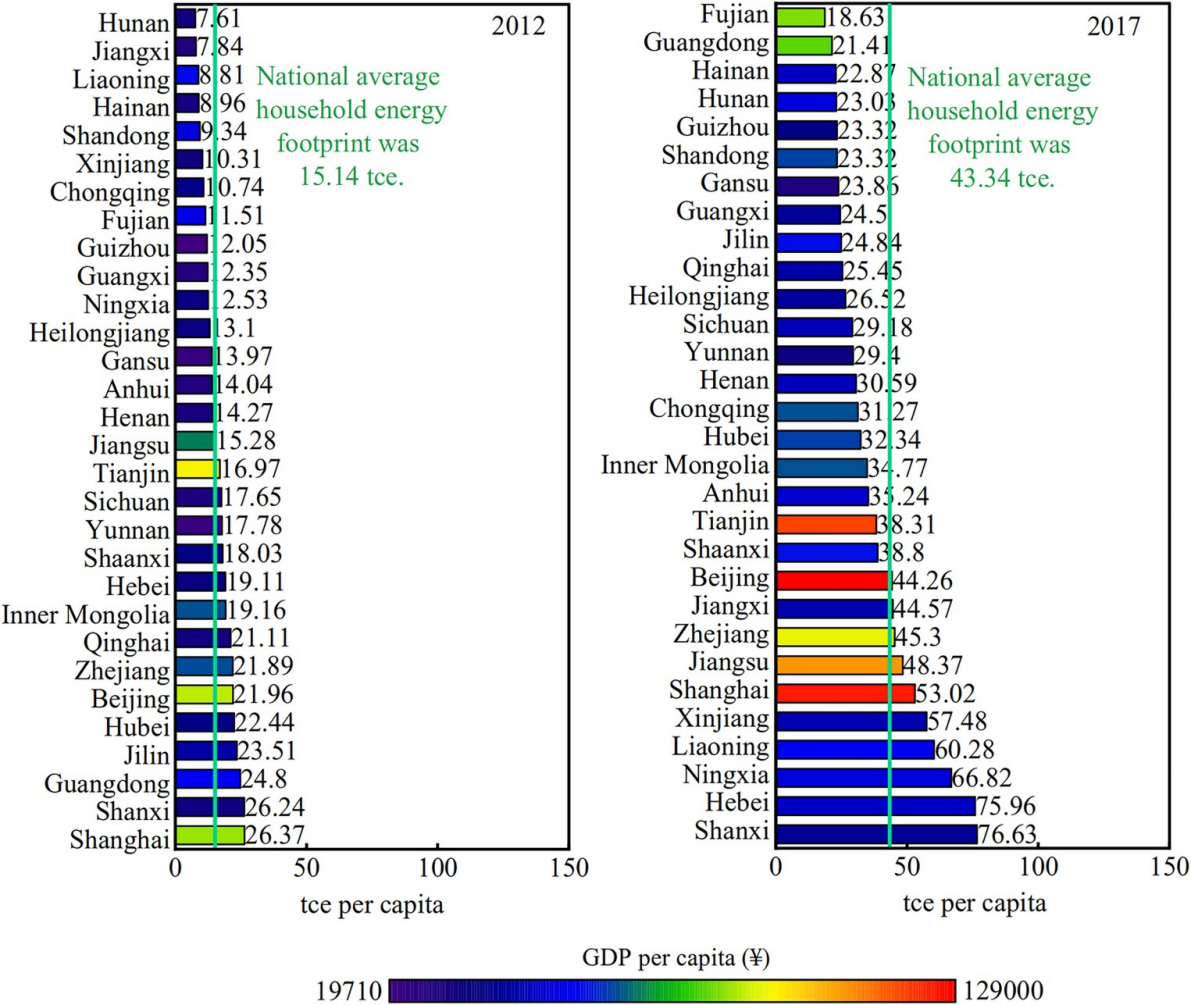
The PCHEF of 30 provinces in China in 2012 and 2017. The colors of these numerical bars correspond to provincial GDP per capita, ranging from red for the richest province to blue for the poorest.

The PCHEF varies considerably across Chinese provinces, with wealthier regions typically having a higher energy footprint than poorer regions. In 2012, two affluent eastern regions (Beijing and Shanghai) had a PCHEF of more than 21 tce (a ton of standard coal equivalent), while some central and western provinces (Hunan, Jiangxi, and Xinjiang) had an energy footprint of fewer than 11 tce (Fig 1). For instance, the PCHEF of households in Shanghai, China’s most significant economic hub, was 26.37 tce, more than twice as high as in Guizhou, the poorer province in the west. In 2017, Shanxi was the province with the highest PCHEF in China (76.63 tce), four times higher than the lowest province (Fujian, 18.63 tce). Shanxi is a significant energy province in China, providing strong energy support for national construction, with a coal-bed methane extraction volume of 12 billion m^3^ in 2017, accounting for more than half of the country. Nevertheless, the energy consumption structure of Shanxi is not reasonable, with a high proportion of coal consumption and a low proportion of non-fossil energy. Between 2012 and 2017, the whole PCHEF in China increased by 186%, from 15.14 to 43.34 tce. The PCHEF in the vast majority of provinces was increasing, and it was growing faster in less developed provinces. On the whole, energy inequality in China was decreasing between 2012 and 2017. This study further investigates whether such convergence exists in China. The results show that the provinces with lower PCHEF in 2012 experienced the largest increase in the PCHEF from 2012 to 2017. After distinguishing between rural and urban households, the convergence calculation results show that urban households display more pronounced convergence in the Gini coefficient of energy footprint.

**Fig 2:**
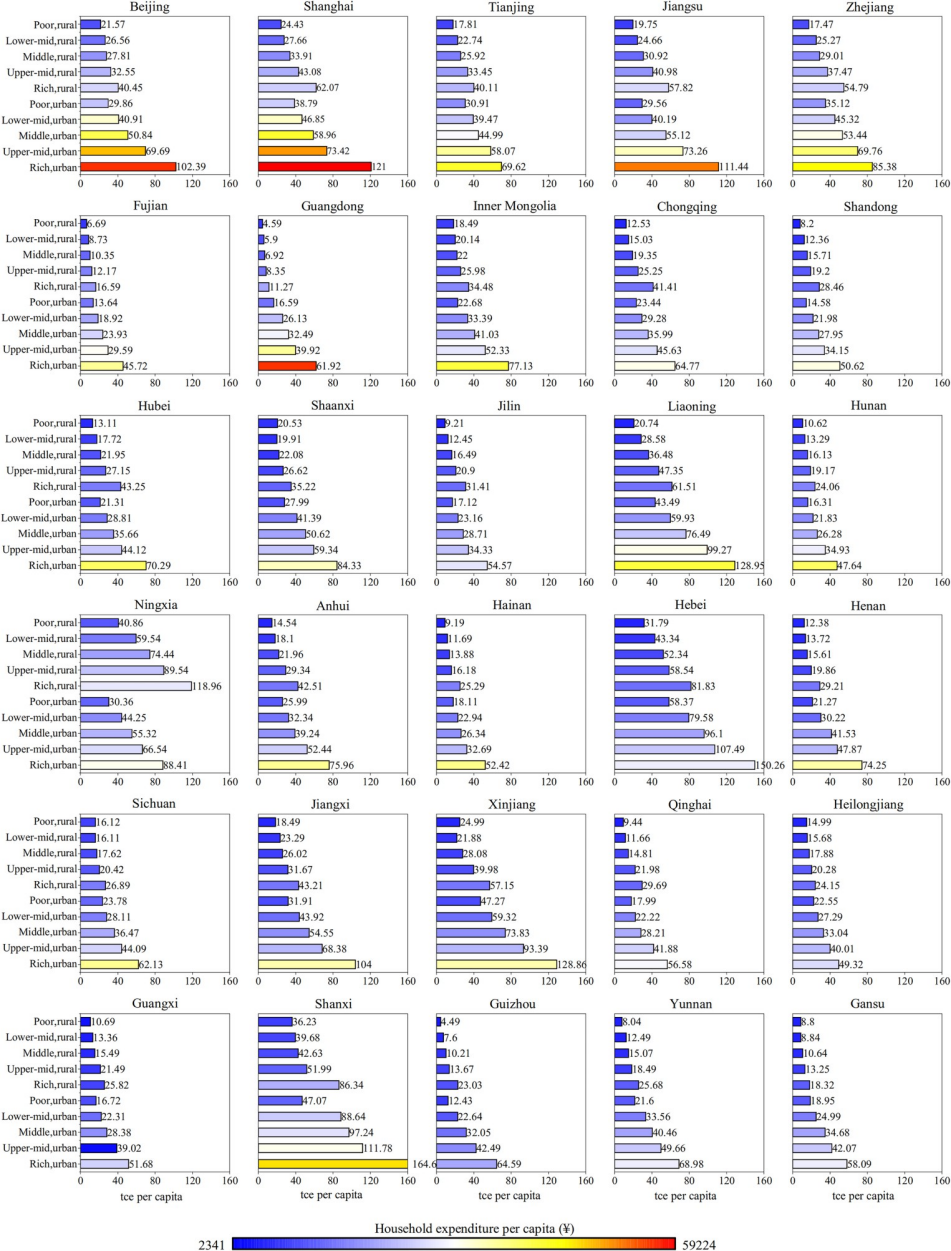
The PCHEF of 10 income groups in China’s 30 provinces in 2017. Bar colors correspond to household consumption expenditure per capita, with the wealthiest group in red and the poorest group in blue (see scale). All provinces are ranked by GDP per capita, from the wealthiest province (Beijing) in the first row, first column, to the poorest province (Gansu) in the sixth row, fifth column.

In 2017, urban residents, who comprised 59% of China’s population, accounted for 66% of the national household energy footprint. The PCHEF of urban residents was 48.82 tce, 1.9 times that of rural residents (25.61 tce). In contrast, the per capita expenditure was 2.7 times higher than that of rural residents. Fig 2 illustrates the PCHEF of 10 income groups in 30 provinces in China in 2017. The energy footprint of urban residents was distinctly higher than that of rural residents in all provinces. The PCHEF between urban and rural residents showed greater differences in the central and western provinces of China. For example, in 2017, Gansu, whose GDP per capita was at the bottom of the country, had a PCHEF for urban residents that was 3.0 times higher than that of rural residents (Fig 2, row 6, column 5). By comparison, the PCHEF of urban residents in Beijing and Shanghai, two of China’s wealthiest regions, was only 1.8 times that of rural residents. But what is more remarkable is that Guangdong province, which owned a higher GDP, had the most enormous difference in the PCHEF between urban and rural residents (up to 4.8 times).

The income groups with the highest PCHEF are primarily located in relatively poor provinces (Table A1 in S1 Appendix). In 2017, the PCHEF of the wealthy urban income groups in Shanxi, Hebei, and Liaoning were 164.60, 150.26, and 128.95 tce, respectively. The per capita consumption expenditure of the top 20 percent of urban households in Shanxi was 46,577 yuan, which was even higher than that of the top 20 percent of urban households in Zhejiang and Tianjin (44,692 yuan and 42,346 yuan). Although the per capita consumption expenditure of the top 20 percent of urban households in Shanxi was almost the same as that in Zhejiang, the PCHEF of the two groups was very different (Fig 2), which was mainly due to the geographical differences between the two provinces. Shanxi, located in North China, has a strong temperate continental climate due to the topography and sea breeze, which requires long-term heating in winter. Zhejiang, located in eastern China, has a subtropical monsoon climate with moderate annual temperatures and no central heating facilities. The high energy consumption of heating homes in the north, contributes to the difference in the energy footprint of households in the North and south. In addition, Shanxi province has a high proportion of the heavy industry, which is one of the essential reasons for its high energy footprint [45].

The income groups with the lowest PCHEF are primarily located in provinces with weaker economic development (Table A2 in S1 Appendix). In 2017, the PCHEF of the lowest income group in Guizhou, Yunnan, Gansu, and Qinghai was lower than 10 tce, which was mainly attributed to their lower household consumption expenditure. The per capita household consumption expenditure in the rural poor income groups in the four provinces was about 5,683 yuan in 2017, only one-third of the overall average for China (18,322 yuan). Whereas two of China’s wealthier coastal provinces, Guangdong and Fujian, had a PCHEF of only 6 tce for their rural low-income groups. The average PCHEF in these two provinces was relatively low, mainly due to the constraints of the dual goals of controlling energy consumption and intensity in the 13th Five-Year Plan [46].

**Fig 3:**
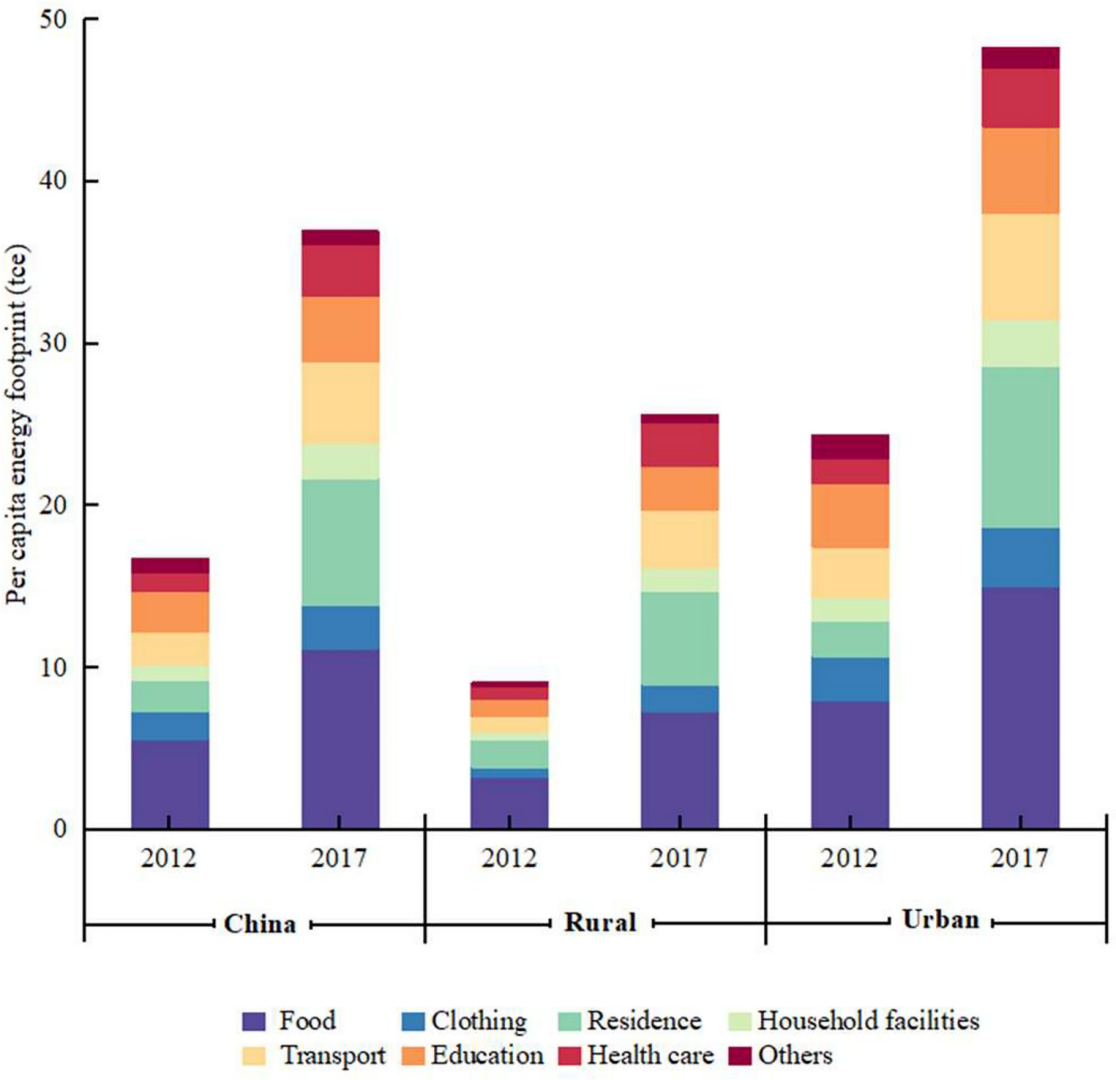
The PCHEF for eight consumption types in urban and rural China in 2012 and 2017.

This paper further divides the consumption expenditure of Chinese households into eight types, and calculates the PCHEF of Chinese urban and rural households for each type of consumption (Fig 3). In 2017, food consumption actuated the largest share of the PCHEF (30%), followed by residence (21%) and transport (14%). The accelerated urbanization in China and the increase in residential buildings and transportation are driving the growth of residents’ PCHEF [47, 48]. On the whole, the PCHEF of China from residential consumption increased by 310.7% from 2012 to 2017, and the energy footprint of residential consumption increased the most among all consumption types. The PCHEF of food, clothing, and education decreased slightly. The consumption type with the largest increase in the PCHEF varies between urban and rural areas. The largest increase in the PCHEF of urban residents was observed for residence and health care, which increased by 362.0 percent and 142.4 percent, respectively. The PCHEF of rural residents saw the largest increase in residence and transportation consumption, which increased by 244.3 percent and 269.8 percent, respectively. For rural households, it is likely that the bulk of income is spent on basic needs such as residence and transportation. In contrast, the increase in demand for high-end consumer goods by urban households is second only to basic needs, such as health care. Although the growth in these consumption categories is slight, urban households consume large amounts and significantly increase their energy footprint [49].

### Equality of household energy footprint

**Fig 4:**
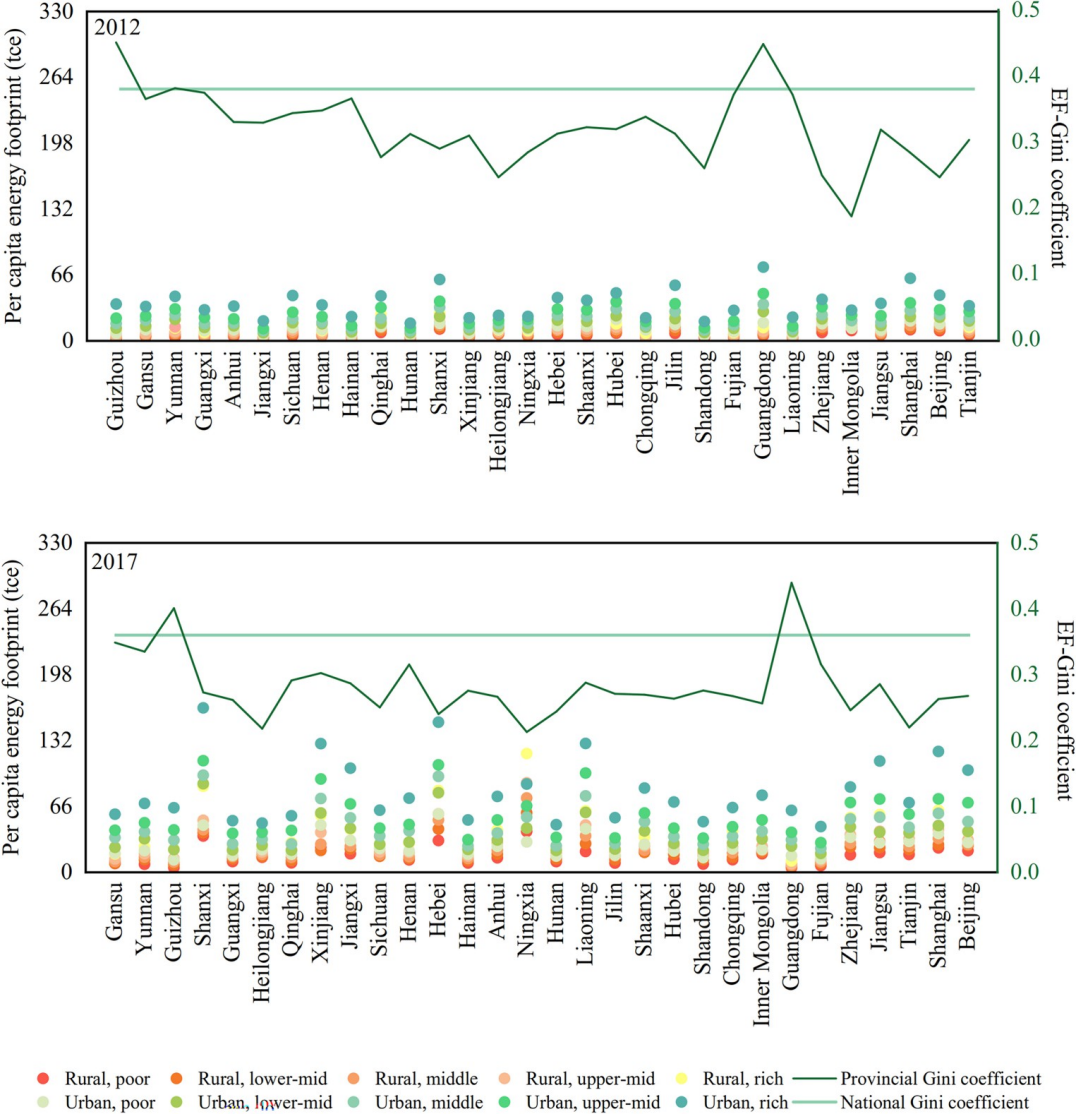
The PCHEF of different income groups and EF-Gini of each province. All provinces are ranked based on GDP per capita, from left to right, from the poorest province with the lowest GDP per capita (Guizhou in 2012, Gansu in 2017) to the highest (Tianjin in 2012, Beijing in 2017).

This study uses the EF-Gini to measure the energy inequality of households, with 0 indicating perfect equality and 1 indicating perfect inequality. Energy inequality in China decreases as the economy grows. At the national level, between 2012 and 2017, the EF-Gini slightly reduced from 0.38 to 0.36 in China (Fig 4), while the officially published income Gini coefficient decreased from 0.34 to 0.31. In 2017, the PCHEF of the top 10% income group accounted for 22% of the national energy footprint, while the PCHEF of the bottom 40% income group accounted for only 24%. At the provincial level, poorer western provinces generally had higher EF-Gini. In 2017, two western provinces (Guizhou and Gansu) had an average Gini coefficient of 0.38. By contrast, three wealthy provinces (Beijing, Shanghai, and Tianjin) had a GDP per capita of more than ¥119,000, while these regions had very low EF-Gini (0.26, 0.27, and 0.22). In particular, although the Gini coefficient of the energy footprint of relatively wealthy Guangdong Province has decreased slightly, its energy footprint is still higher than that of the whole country, which reflects the prominent problem of unbalanced development in the region. The higher Gini coefficient of energy footprint is usually greatly related to the differences in household consumption patterns and low-carbon life concepts of different income groups, which may be one of the reasons for the serious energy inequality problem in Guangdong province compared with other wealthy provinces [50]. With the exception of Inner Mongolia and Beijing, most provinces saw their EF-Gini decline between 2012 and 2017 (Fig A2 in S1 Appendix), and the less developed western provinces saw their EF-Gini decline faster. For instance, the EF-Gini of Guangxi and Sichuan decreased by 0.11 and 0.09, respectively. It can be seen from the calculation results of the Gini coefficient of energy footprint that the inequality of China’s household energy footprint is decreasing with China’s economic growth in both time and space.

**Fig 5:**
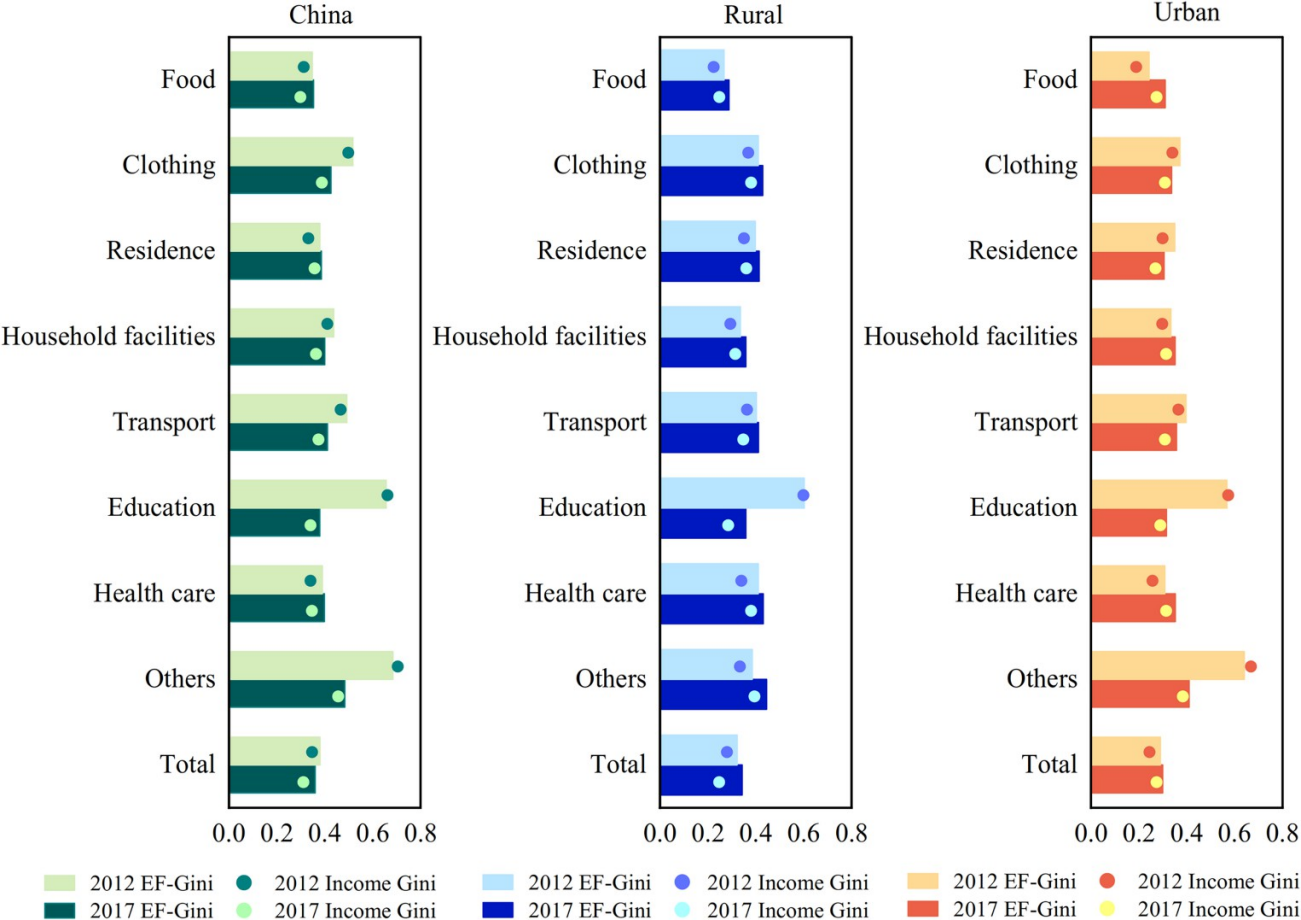
EF-Gini and income Gini coefficient for overall, rural, and urban China in 2012 and 2017. The EF-Gini and income Gini coefficient are divided into eight consumption expenditure categories.

From 2012 to 2017, the EF-Gini of the overall energy footprint at the national level decreased, while the EF-Gini of the internal energy footprint of urban and rural areas increased, indicating that the gap of energy inequality between urban and rural residents was narrowing (Fig 5). Except for food, housing and health care, the EF-Gini of all other consumption categories decreased nationwide, with education spending seeing the biggest drop. The EF-Gini of different consumption types in different provinces also differ during the same period (Tables A4 and A5 in S1 Appendix). The above results reflect that energy inequality is closely correlated with consumption quantity (Table A3 in S1 Appendix) and consumption patterns. Residence and health care consumption are the main factors contributing to the differences in energy footprints across groups. Thus, the size and inequality of the energy footprint can be reduced by improving housing conditions, developing energy-saving habits, and balancing health care provision [51]. As China’s real estate market and health care sector continue to develop and improve, the equality of China’s household energy footprint will further improve [52, 53].

## Conclusion

Household energy footprints are dramatically unequal within and across provinces in China.

First, the PCHEF of urban residents is 1.9 times that of rural residents. Urban residents have a superior standard of living and consume more energy-intensive household appliances and transportation than rural residents, which drives a more extensive energy footprint. Between 2012 and 2017, the EF-Gini rose slightly in urban (0.2898–0.2992) and rural (0.3222–0.3430) areas in China. However, the Gini coefficient of China’s overall household energy footprint is declining (0.3802–0.3600). The above conclusions suggest that the gap in the household energy footprint between urban and rural residents in China is narrowing with the acceleration of urbanization.

Second, there are significant variations in the PCHEF among China’s provinces. On the whole, the PCHEF of the wealthier coastal provinces is higher than that of the poorer inland regions. From the perspective of production, the advantage of natural resource endowment makes the industrial structure of the western region biased towards energy-intensive heavy industry, which results in higher energy intensity. From the perspective of consumption, the spill-over effect of products has led to the flow of more energy-intensive products produced in the west to the east, causing the energy consumption spilled from the west to the east to be much larger than the internal consumption. With the deepening of reform and opening up, China has begun to focus on coordinated regional development and made great efforts to narrow the economic development gap between the east and the west. The Western Development Strategy and the Strategy for Promoting the Rise of the Central Region have been proposed, aiming to promote the rapid development of the central and western regions. At last, the gap between the energy footprint of eastern and western China decreased significantly between 2012 and 2017, and most of the provinces with the higher PCHEF in 2017 were located in the central and western regions.

Third, there are significant differences in the scale of the energy footprint between income groups in China. While the PCHEF of the richest groups in Shanxi and Hebei is as high as 150 tce, the PCHEF of the poorest groups in Guizhou, Yunnan, and Gansu is only 6.9 tce. Increasing income actuates the growth of residential consumption, which bringing about an increase in the corresponding consumption energy footprint. In 2017, the PCHEF of the top 10% income group accounted for 22% of the national energy footprint, while the PCHEF of the bottom 40% income group accounted for only 24%.

Based on the findings discussed in this paper, we conclude that energy inequality in China has been decreasing with economic growth. Reducing energy inequality can struggle away with two aspects: raising the income of the poor to gradually narrow the income gap and changing the lifestyles of the rich to reduce the energy intensity of their consumption patterns. The government can accomplish the dual goals of poverty reduction and energy structure optimization by carrying out poverty reduction projects, especially those related to the energy economy. In addition, the government can also promote the adjustment of the energy structure by establishing an energy regulatory mechanism, adjusting energy taxation and promoting the construction of low-carbon public infrastructure. Meanwhile, it is necessary to guide high-income groups follow a more green and low-carbon lifestyle, change their energy consumption structure and increase the proportion of clean energy consumption.

Another meaningful measure to eliminate energy footprint inequality hinges on closing the gap between the energy footprint of urban and rural residents. Rural residents in some regions primarily burn briquets and straw to obtain fuel for cooking and heating, etc. Therefore, a key issue in reducing the inequality in household energy footprint between urban and rural areas is to adjust the rural energy utilization structure and promote rural residents to use more efficient and clean cooking energy such as electricity and LPG instead of traditional fuels. In the next place, developing and sharing green and low-carbon heating technologies is vital to optimize the energy consumption of rural residents. Therefore, the Chinese government should continue to deepen reform, actively promote the optimization of industrial structure and energy structure, improve energy efficiency, and increase the penetration rate of renewable energy (wind, solar and biomass).

## Supporting information

S1 Appendix (DOCX)

S1 File (RAR)
