# Supplementary material for: Economic growth and household energy footprint inequality in China
Source: PLoS One. 2023 Mar 1;18(3):e0282300. doi: 10.1371/journal.pone.0282300 (PMC9977031; doi:10.1371/journal.pone.0282300)
Supplement: S1 Appendix — (DOCX) [file pone.0282300.s001.docx]

**Appendix A**

**Figure A1. Change in Gini coefficient of energy footprint for overall, rural, and urban areas in 30 Chinese provinces between 2012 and 2017.**

**Figure A2. The PCHEF of 10 income groups in 30 Chinese provinces in 2012. Bar colors correspond to household consumption expenditure per capita, with the wealthiest group in red and the poorest group in blue (see scale). All provinces are ranked by GDP per capita in 2017, from the wealthiest province (Beijing), located in the first row, first column, to the poorest province (Gansu), in the sixth row, fifth column.**


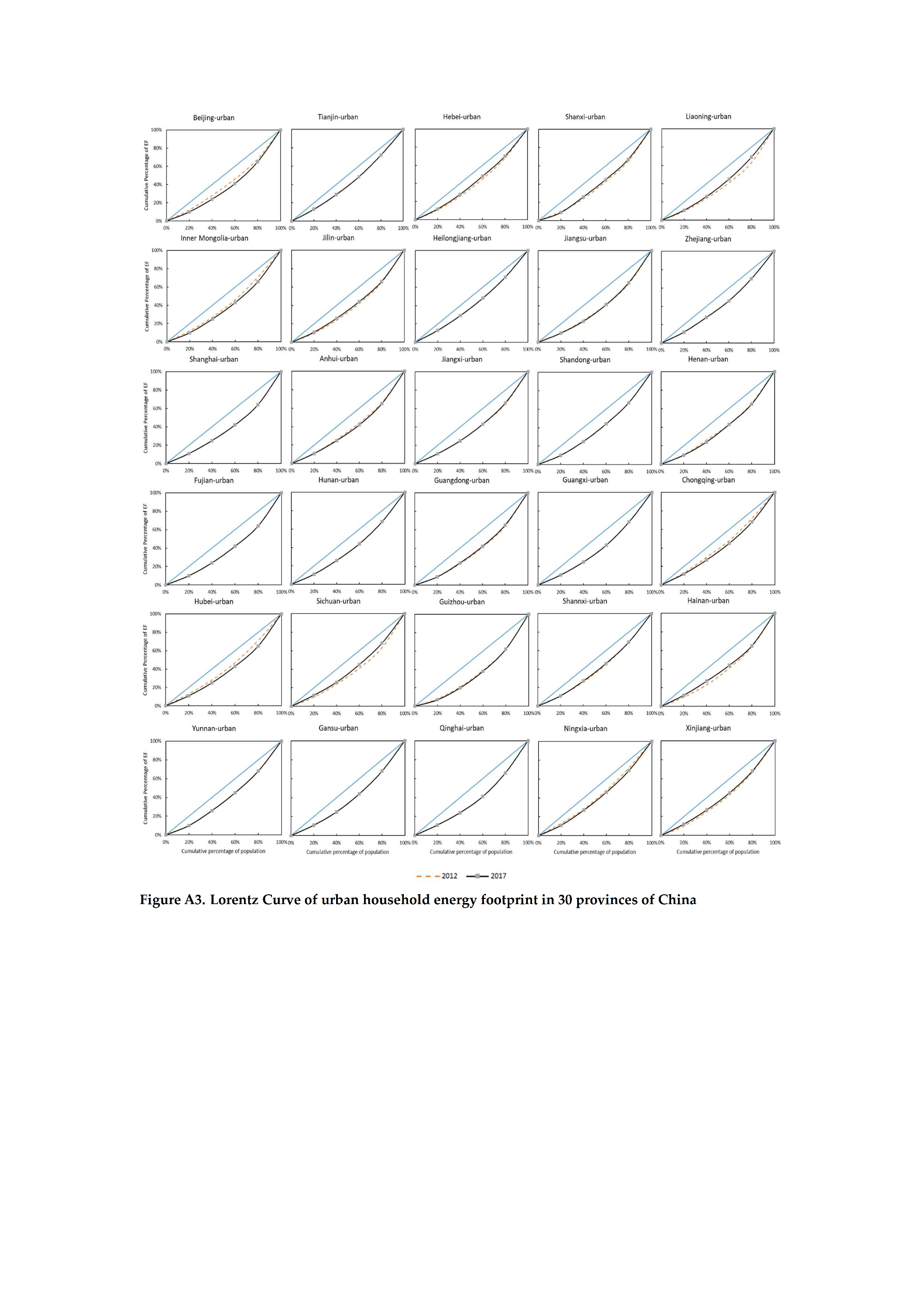


**Figure A3. Lorentz Curve of urban household energy footprint in 30 provinces of China**


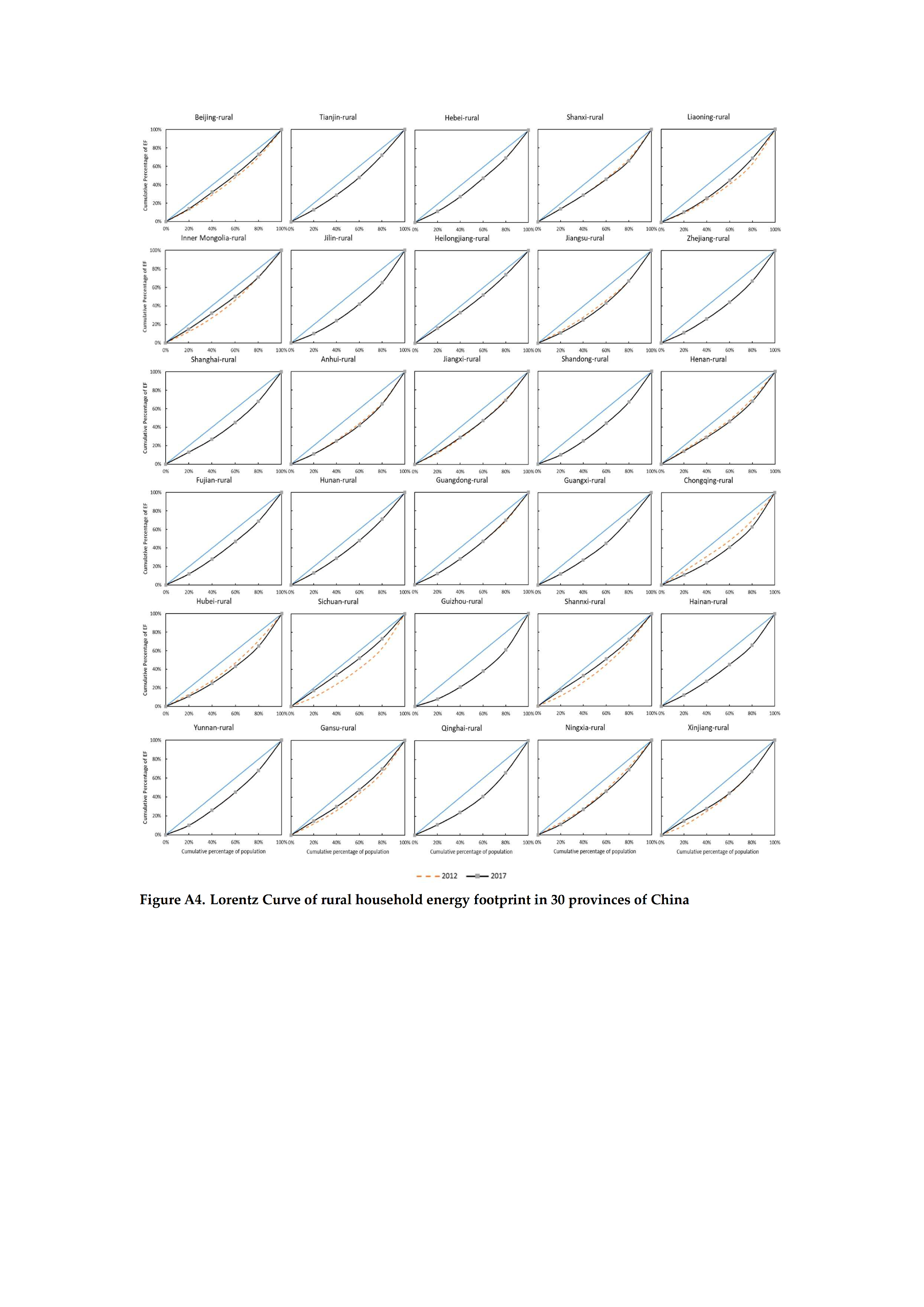


**Figure A4. Lorentz Curve of rural household energy footprint in 30 provinces of China**

**Table A1. Top 20 income groups with the highest PCHEF in China, 2017.**

| **No.** | **Provinces** | **Urban/Rural** | **Income groups** | **Energy footprint (tce)** |
| --- | --- | --- | --- | --- |
| 1 | Shanxi | Urban | Rich | 164.60 |
| 2 | Hebei | Urban | Rich | 150.26 |
| 3 | Liaoning | Urban | Rich | 128.95 |
| 4 | Xinjiang | Urban | Rich | 128.86 |
| 5 | Shanghai | Urban | Rich | 121.00 |
| 6 | Shanxi | Urban | Upper-mid | 111.78 |
| 7 | Jiangsu | Urban | Rich | 111.44 |
| 8 | Hebei | Urban | Upper-mid | 107.49 |
| 9 | Jiangxi | Urban | Rich | 104.00 |
| 10 | Beijing | Urban | Rich | 102.39 |
| 11 | Liaoning | Urban | Upper-mid | 99.27 |
| 12 | Shanxi | Urban | Middle | 97.24 |
| 13 | Hebei | Urban | Middle | 96.10 |
| 14 | Xinjiang | Urban | Upper-mid | 93.39 |
| 15 | Shanxi | Urban | Lower-mid | 88.64 |
| 16 | Ningxia | Urban | Rich | 88.41 |
| 17 | Zhejiang | Urban | Rich | 85.38 |
| 18 | Shaanxi | Urban | Rich | 84.33 |
| 19 | Hebei | Urban | Lower-mid | 79.58 |
| 20 | Inner Mongolia | Urban | Rich | 77.13 |

**Table A2.** **Bottom 20 income groups with the lowest PCHEF in China, 2017.**

| **No.** | **Provinces** | **Urban/Rural** | **Income groups** | **Energy footprint (tce)** |
| --- | --- | --- | --- | --- |
| 1 | Guizhou | Rural | Poor | 4.49 |
| 2 | Guangdong | Rural | Poor | 4.59 |
| 3 | Guangdong | Rural | Lower-mid | 5.90 |
| 4 | Fujian | Rural | Poor | 6.69 |
| 5 | Guangdong | Rural | Middle | 6.92 |
| 6 | Guizhou | Rural | Lower-mid | 7.60 |
| 7 | Yunnan | Rural | Poor | 8.04 |
| 8 | Shandong | Rural | Poor | 8.20 |
| 9 | Guangdong | Rural | Upper-mid | 8.35 |
| 10 | Gansu | Rural | Poor | 8.80 |
| 11 | Gansu | Rural | Lower-mid | 8.84 |
| 12 | Hainan | Rural | Poor | 9.19 |
| 13 | Jilin | Rural | Poor | 9.21 |
| 14 | Qinghai | Rural | Poor | 9.44 |
| 15 | Guizhou | Rural | Middle | 10.21 |
| 16 | Fujian | Rural | Lower-mid | 10.35 |
| 17 | Hunan | Rural | Poor | 10.62 |
| 18 | Gansu | Rural | Middle | 10.64 |
| 19 | Guangxi | Rural | Poor | 10.69 |
| 20 | Guangdong | Rural | Rich | 11.27 |

**Table A3.** **The** **PCHEF of eight consumption types in 30 provinces of China in 2017.**

| **Region** | **Provinces** | **Food** | **Clothing** | **Residence** | **Household Facilities** | **Transport** | **Education** | **Health Care** | **Others** |
| --- | --- | --- | --- | --- | --- | --- | --- | --- | --- |
|  | Beijing | 7.37 | 1.64 | 8.83 | 2.55 | 4.35 | 2.06 | 2.68 | 0.33 |
|  | Tianjin | 8.51 | 1.87 | 5.75 | 1.73 | 4.80 | 2.38 | 2.51 | 0.45 |
|  | Hebei | 14.32 | 3.48 | 12.10 | 3.39 | 8.59 | 5.16 | 5.41 | 1.06 |
|  | Shanxi | 9.16 | 2.32 | 7.64 | 1.58 | 4.18 | 4.41 | 3.81 | 0.61 |
|  | Inner Mongolia | 6.71 | 1.68 | 4.38 | 1.04 | 4.10 | 2.23 | 2.57 | 0.52 |
|  | Liaoning | 10.35 | 2.52 | 7.93 | 1.86 | 6.33 | 4.66 | 4.54 | 0.74 |
|  | Jilin | 4.97 | 1.21 | 3.20 | 0.74 | 2.84 | 2.28 | 2.44 | 0.39 |
|  | Heilongjiang | 4.91 | 1.29 | 3.04 | 0.76 | 2.50 | 2.04 | 12.42 | 0.40 |
|  | Shanghai | 22.01 | 3.85 | 31.47 | 20.35 | 9.04 | 10.69 | 5.78 | 2.68 |
|  | Jiangsu | 10.02 | 1.99 | 7.59 | 2.13 | 5.88 | 3.19 | 3.13 | 0.89 |
|  | Zhejiang | 10.11 | 1.73 | 7.92 | 1.53 | 5.73 | 2.88 | 2.45 | 0.48 |
|  | Anhui | 8.38 | 1.30 | 5.92 | 1.34 | 3.16 | 2.43 | 2.32 | 0.42 |
|  | Fujian | 4.02 | 0.48 | 2.84 | 0.54 | 1.19 | 0.93 | 0.70 | 0.24 |
|  | Jiangxi | 9.54 | 1.48 | 7.38 | 1.57 | 3.16 | 2.86 | 2.56 | 0.49 |
| Rural | Shandong | 4.71 | 0.95 | 3.20 | 1.13 | 2.85 | 1.86 | 1.86 | 0.25 |
|  | Henan | 4.84 | 1.39 | 3.99 | 1.29 | 2.48 | 1.97 | 1.87 | 0.32 |
|  | Hubei | 6.98 | 0.68 | 5.33 | 1.52 | 2.94 | 2.80 | 3.10 | 0.65 |
|  | Hunan | 5.08 | 0.77 | 3.72 | 0.94 | 1.80 | 2.47 | 1.70 | 0.24 |
|  | Guangdong | 2.96 | 0.26 | 1.62 | 0.41 | 0.82 | 0.66 | 0.51 | 0.16 |
|  | Guangxi | 5.52 | 0.53 | 3.98 | 0.91 | 2.41 | 2.08 | 1.69 | 0.27 |
|  | Hainan | 6.36 | 0.52 | 2.92 | 1.69 | 1.00 | 1.86 | 0.60 | 0.28 |
|  | Chongqing | 6.54 | 0.98 | 3.22 | 1.23 | 2.19 | 2.01 | 1.45 | 0.30 |
|  | Sichuan | 7.30 | 7.43 | 3.76 | 1.37 | 2.44 | 1.48 | 1.93 | 0.39 |
|  | Guizhou | 4.49 | 0.59 | 2.40 | 0.64 | 1.54 | 1.13 | 0.86 | 0.17 |
|  | Yunnan | 3.97 | 0.72 | 3.29 | 1.04 | 2.95 | 2.37 | 1.49 | 0.21 |
|  | Shaanxi | 6.43 | 1.41 | 5.74 | 1.56 | 3.03 | 2.84 | 3.38 | 0.48 |
|  | Gansu | 3.60 | 0.75 | 1.81 | 0.72 | 1.54 | 1.56 | 1.36 | 0.21 |
|  | Qinghai | 5.16 | 1.67 | 2.99 | 0.79 | 3.04 | 1.68 | 2.24 | 0.50 |
|  | Ningxia | 8.46 | 2.53 | 6.54 | 1.97 | 6.15 | 4.29 | 3.37 | 0.68 |
|  | Xinjiang | 10.40 | 2.77 | 6.57 | 1.62 | 5.76 | 2.93 | 3.87 | 0.49 |
|  | Beijing | 11.44 | 3.53 | 19.59 | 3.85 | 7.89 | 6.32 | 4.48 | 1.67 |
|  | Tianjin | 11.59 | 3.31 | 10.44 | 2.62 | 6.11 | 4.95 | 4.37 | 1.37 |
|  | Hebei | 24.19 | 8.06 | 24.10 | 7.09 | 13.96 | 10.37 | 8.29 | 2.28 |
|  | Shanxi | 68.22 | 6.53 | 14.37 | 4.19 | 9.84 | 9.42 | 6.35 | 1.77 |
|  | Inner Mongolia | 12.35 | 4.97 | 7.84 | 3.22 | 6.78 | 5.03 | 2.78 | 2.34 |
|  | Liaoning | 22.35 | 6.99 | 14.49 | 4.98 | 12.19 | 10.14 | 7.69 | 2.80 |
|  | Jilin | 8.08 | 3.10 | 5.96 | 1.78 | 4.45 | 3.80 | 3.42 | 0.99 |
|  | Heilongjiang | 9.87 | 3.33 | 11.54 | 0.94 | 4.49 | 4.03 | 3.52 | 0.96 |
|  | Shanghai | 9.81 | 1.50 | 7.62 | 1.52 | 3.85 | 1.97 | 2.35 | 0.57 |
|  | Jiangsu | 16.80 | 4.10 | 15.13 | 3.83 | 8.91 | 7.79 | 3.53 | 1.80 |
|  | Zhejiang | 16.47 | 3.47 | 14.61 | 2.74 | 8.93 | 6.37 | 3.55 | 1.21 |
|  | Anhui | 14.34 | 3.39 | 9.16 | 2.64 | 6.54 | 5.13 | 2.82 | 1.17 |
|  | Fujian | 8.51 | 1.46 | 6.93 | 1.51 | 3.52 | 2.75 | 1.25 | 0.63 |
|  | Jiangxi | 18.56 | 4.84 | 14.42 | 3.84 | 6.97 | 6.98 | 3.35 | 1.63 |
| Urban | Shandong | 7.84 | 13.09 | 6.34 | 2.28 | 4.36 | 3.41 | 2.35 | 0.71 |
|  | Henan | 11.35 | 3.92 | 9.44 | 3.51 | 5.04 | 4.86 | 3.65 | 1.25 |
|  | Hubei | 12.17 | 2.93 | 8.80 | 2.46 | 4.03 | 4.53 | 4.14 | 0.98 |
|  | Hunan | 8.26 | 2.14 | 5.54 | 1.93 | 3.72 | 5.01 | 2.16 | 0.63 |
|  | Guangdong | 11.21 | 1.89 | 8.41 | 2.12 | 5.07 | 3.87 | 1.74 | 1.09 |
|  | Guangxi | 10.41 | 1.57 | 6.75 | 1.89 | 4.57 | 3.72 | 2.15 | 0.61 |
|  | Hainan | 11.16 | 1.39 | 5.74 | 1.69 | 4.21 | 3.38 | 2.30 | 0.65 |
|  | Chongqing | 12.78 | 3.41 | 6.93 | 2.79 | 5.24 | 4.42 | 3.29 | 0.86 |
|  | Sichuan | 12.81 | 3.07 | 6.91 | 2.50 | 5.73 | 3.95 | 2.86 | 1.10 |
|  | Guizhou | 11.49 | 2.68 | 6.08 | 2.32 | 4.83 | 4.67 | 2.00 | 0.69 |
|  | Yunnan | 11.94 | 2.54 | 8.72 | 2.65 | 6.98 | 5.33 | 3.88 | 0.98 |
|  | Shaanxi | 14.82 | 4.21 | 9.77 | 3.91 | 6.34 | 6.67 | 5.62 | 1.40 |
|  | Gansu | 10.31 | 3.31 | 6.56 | 2.38 | 5.18 | 4.11 | 2.99 | 1.89 |
|  | Qinghai | 9.34 | 2.90 | 5.79 | 1.99 | 5.31 | 4.16 | 3.02 | 0.93 |
|  | Ningxia | 13.75 | 5.14 | 10.17 | 3.58 | 10.55 | 7.70 | 4.78 | 1.56 |
|  | Xinjiang | 22.36 | 7.18 | 13.85 | 5.76 | 12.43 | 9.18 | 7.48 | 2.29 |

**Table A4.** **EF-Gini of 8 categories of household consumption expenditure in 30 provinces of China in 2012.**

| **No** | **Provinces** | **Food** | **Clothing** | **Residence** | **Household Facilities** | **Transport** | **Education** | **Health Care** | **Others** |
| --- | --- | --- | --- | --- | --- | --- | --- | --- | --- |
| 1 | Beijing | 0.20 | 0.33 | 0.16 | 0.27 | 0.34 | 0.35 | 0.15 | 0.43 |
| 2 | Tianjin | 0.28 | 0.333 | 0.17 | 0.335 | 0.38 | 0.371 | 0.25 | 0.44 |
| 3 | Hebei | 0.30 | 0.39 | 0.20 | 0.319 | 0.34 | 0.37 | 0.27 | 0.33 |
| 4 | Shanxi | 0.26 | 0.36 | 0.19 | 0.37 | 0.36 | 0.33 | 0.36 | 0.41 |
| 5 | Inner Mongolia | 0.24 | 0.42 | 0.29 | 0.444 | 0.30 | 0.82 | 0.26 | 0.41 |
| 6 | Liaoning | 0.31 | 0.45 | 0.22 | 0.48 | 0.39 | 0.50 | 0.35 | 0.51 |
| 7 | Jilin | 0.23 | 0.43 | 0.25 | 0.42 | 0.43 | 0.33 | 0.27 | 0.43 |
| 8 | Heilongjiang | 0.23 | 0.34 | 0.16 | 0.31 | 0.33 | 0.24 | 0.50 | 0.32 |
| 9 | Shanghai | 0.20 | 0.36 | 0.28 | 0.39 | 0.43 | 0.38 | 0.14 | 0.52 |
| 10 | Jiangsu | 0.27 | 0.44 | 0.21 | 0.38 | 0.39 | 0.38 | 0.28 | 0.47 |
| 11 | Zhejiang | 0.19 | 0.31 | 0.20 | 0.25 | 0.38 | 0.36 | 0.17 | 0.39 |
| 12 | Anhui | 0.28 | 0.75 | 0.65 | 0.32 | 0.43 | 0.44 | 0.34 | 0.44 |
| 13 | Fujian | 0.30 | 0.44 | 0.27 | 0.42 | 0.51 | 0.44 | 0.32 | 0.49 |
| 14 | Jiangxi | 0.27 | 0.47 | 0.24 | 0.41 | 0.46 | 0.47 | 0.23 | 0.47 |
| 15 | Shandong | 0.25 | 0.41 | 0.23 | 0.36 | 0.37 | 0.37 | 0.30 | 0.47 |
| 16 | Henan | 0.32 | 0.44 | 0.26 | 0.47 | 0.53 | 0.46 | 0.34 | 0.50 |
| 17 | Hubei | 0.31 | 0.45 | 0.16 | 0.34 | 0.41 | 0.43 | 0.23 | 0.31 |
| 18 | Hunan | 0.25 | 0.44 | 0.21 | 0.38 | 0.43 | 0.40 | 0.27 | 0.45 |
| 19 | Guangdong | 0.36 | 0.51 | 0.33 | 0.48 | 0.55 | 0.55 | 0.39 | 0.50 |
| 20 | Guangxi | 0.31 | 0.53 | 0.26 | 0.47 | 0.51 | 0.51 | 0.30 | 0.49 |
| 21 | Hainan | 0.33 | 0.49 | 0.26 | 0.43 | 0.50 | 0.36 | 0.36 | 0.50 |
| 22 | Chongqing | 0.32 | 0.44 | 0.25 | 0.33 | 0.38 | 0.38 | 0.24 | 0.89 |
| 23 | Sichuan | 0.26 | 0.45 | 0.26 | 0.40 | 0.49 | 0.46 | 0.24 | 0.52 |
| 24 | Guizhou | 0.43 | 0.53 | 0.32 | 0.48 | 0.51 | 0.53 | 0.40 | 0.50 |
| 25 | Yunnan | 0.32 | 0.51 | 0.27 | 0.41 | 0.50 | 0.42 | 0.37 | 0.56 |
| 26 | Shaanxi | 0.33 | 0.44 | 0.18 | 0.37 | 0.40 | 0.40 | 0.26 | 0.46 |
| 27 | Gansu | 0.32 | 0.46 | 0.27 | 0.40 | 0.41 | 0.45 | 0.33 | 0.50 |
| 28 | Qinghai | 0.27 | 0.39 | 0.17 | 0.361 | 0.43 | 0.42 | 0.18 | 0.51 |
| 29 | Ningxia | 0.31 | 0.38 | 0.09 | 0.30 | 0.37 | 0.37 | 0.25 | 0.36 |
| 30 | Xinjiang | 0.29 | 0.41 | 0.23 | 0.46 | 0.36 | 0.45 | 0.31 | 0.48 |
| National level | | 0.35 | 0.52 | 0.38 | 0.44 | 0.49 | 0.66 | 0.39 | 0.68 |

**Table A5.** **EF-Gini for 8 categories of household consumption expenditure in 30 provinces of China in 2017.**

| **No** | **Provinces** | **Food** | **Clothing** | **Residence** | **Household Facilities** | **Transport** | **Education** | **Health Care** | **Others** |
| --- | --- | --- | --- | --- | --- | --- | --- | --- | --- |
| 1 | Beijing | 0.18 | 0.30 | 0.31 | 0.28 | 0.28 | 0.36 | 0.23 | 0.49 |
| 2 | Tianjin | 0.20 | 0.26 | 0.19 | 0.23 | 0.26 | 0.29 | 0.20 | 0.38 |
| 3 | Hebei | 0.23 | 0.29 | 0.26 | 0.27 | 0.22 | 0.26 | 0.21 | 0.27 |
| 4 | Shanxi | 0.48 | 0.331 | 0.26 | 0.35 | 0.31 | 0.24 | 0.36 | 0.39 |
| 5 | Inner Mongolia | 0.23 | 0.37 | 0.25 | 0.378 | 0.274 | 0.19 | 0.16 | 0.53 |
| 6 | Liaoning | 0.27 | 0.36 | 0.26 | 0.36 | 0.30 | 0.28 | 0.28 | 0.44 |
| 7 | Jilin | 0.21 | 0.36 | 0.26 | 0.38 | 0.35 | 0.23 | 0.25 | 0.41 |
| 8 | Heilongjiang | 0.22 | 0.29 | 0.34 | 0.15 | 0.30 | 0.22 | 0.55 | 0.30 |
| 9 | Shanghai | 0.24 | 0.35 | 0.46 | 0.37 | 0.42 | 0.43 | 0.27 | 0.45 |
| 10 | Jiangsu | 0.24 | 0.32 | 0.31 | 0.30 | 0.30 | 0.34 | 0.26 | 0.37 |
| 11 | Zhejiang | 0.19 | 0.28 | 0.23 | 0.26 | 0.33 | 0.31 | 0.16 | 0.37 |
| 12 | Anhui | 0.22 | 0.361 | 0.22 | 0.29 | 0.39 | 0.27 | 0.28 | 0.45 |
| 13 | Fujian | 0.25 | 0.37 | 0.32 | 0.37 | 0.43 | 0.35 | 0.24 | 0.38 |
| 14 | Jiangxi | 0.24 | 0.38 | 0.27 | 0.36 | 0.37 | 0.29 | 0.26 | 0.45 |
| 15 | Shandong | 0.21 | 0.34 | 0.29 | 0.33 | 0.33 | 0.30 | 0.29 | 0.40 |
| 16 | Henan | 0.29 | 0.34 | 0.33 | 0.36 | 0.32 | 0.29 | 0.34 | 0.45 |
| 17 | Hubei | 0.23 | 0.44 | 0.27 | 0.33 | 0.27 | 0.25 | 0.31 | 0.31 |
| 18 | Hunan | 0.20 | 0.35 | 0.27 | 0.32 | 0.30 | 0.25 | 0.22 | 0.40 |
| 19 | Guangdong | 0.37 | 0.51 | 0.47 | 0.47 | 0.49 | 0.47 | 0.37 | 0.53 |
| 20 | Guangxi | 0.21 | 0.38 | 0.29 | 0.34 | 0.37 | 0.26 | 0.16 | 0.37 |
| 21 | Hainan | 0.22 | 0.40 | 0.27 | 0.29 | 0.42 | 0.25 | 0.44 | 0.42 |
| 22 | Chongqing | 0.23 | 0.38 | 0.27 | 0.31 | 0.37 | 0.25 | 0.29 | 0.41 |
| 23 | Sichuan | 0.21 | 0.32 | 0.25 | 0.27 | 0.34 | 0.33 | 0.26 | 0.37 |
| 24 | Guizhou | 0.38 | 0.46 | 0.38 | 0.43 | 0.41 | 0.45 | 0.36 | 0.45 |
| 25 | Yunnan | 0.32 | 0.39 | 0.36 | 0.38 | 0.35 | 0.35 | 0.31 | 0.51 |
| 26 | Shaanxi | 0.26 | 0.33 | 0.22 | 0.34 | 0.33 | 0.26 | 0.26 | 0.39 |
| 27 | Gansu | 0.32 | 0.42 | 0.39 | 0.38 | 0.40 | 0.35 | 0.29 | 0.47 |
| 28 | Qinghai | 0.24 | 0.36 | 0.25 | 0.33 | 0.42 | 0.38 | 0.17 | 0.46 |
| 29 | Ningxia | 0.19 | 0.28 | 0.21 | 0.28 | 0.29 | 0.25 | 0.27 | 0.43 |
| 30 | Xinjiang | 0.27 | 0.32 | 0.27 | 0.42 | 0.30 | 0.33 | 0.33 | 0.48 |
| National level | | 0.35 | 0.43 | 0.39 | 0.39 | 0.41 | 0.38 | 0.39 | 0.48 |
